# Molecular Mechanisms Underpinning Astaxanthin-Induced Body Coloration in the *Lutjanus erythropterus* Revealed by Phenotypic, Physiological and Transcriptomic Analyses

**DOI:** 10.3390/ani15223257

**Published:** 2025-11-10

**Authors:** Lei Song, Zizhao Chen, Zhuoxin Lai, Wenjun Feng, Zhongduo Wang, Yusong Guo

**Affiliations:** 1 Key Laboratory of South China Sea Aquatic Economic Animal Cultivation and Breeding, College of Fisheries, Guangdong Ocean University, Zhanjiang 524088, China; 2 Key Laboratory of Aquatic Animal Disease Prevention and Control and Healthy Aquaculture of Guangdong Province, Zhanjiang 524088, China

**Keywords:** *Lutjanus erythropterus*, astaxanthin, body coloration, comparative transcriptomics

## Abstract

Astaxanthin has attracted considerable interest, owing to its potent antioxidant and pigmentation properties. To investigate its effects of astaxanthin on body color variation in *Lutjanus erythropterus*, fish were divided into a control group and a treatment group fed an astaxanthin-supplemented diet. Body color parameters, growth performance, and liver antioxidant enzyme activities were measured at the end of the experiment. Tissues, including skin, intestine, liver, and blood, were subsequently collected for transcriptome sequencing. The results demonstrate that the astaxanthin-treatment group exhibited significantly enhanced body coloration alongside improved body length, body weight, and specific growth rate compared to the control group (*p* < 0.05). Specifically regarding the red–green value (a*), the treatment group showed significantly higher values on the ventral skin, dorsal skin, and gill cover (*p* < 0.05). The yellow–blue (b*) and lightness (L*) values were also significantly elevated in the ventral skin and gill cover (*p* < 0.05), although no significant differences were observed in the dorsal skin (*p* > 0.05). The skin was identified as the tissue with the highest total carotenoid content. Astaxanthin supplementation enhanced liver antioxidant capacity, evidenced by significantly elevated total superoxide dismutase (T-SOD) activity and significantly reduced malondialdehyde (MDA) levels in the treatment group (*p* < 0.05). Catalase (CAT) activity did not differ significantly between groups (*p* > 0.05). Transcriptomic analysis identified several coloration-associated genes, such as bco1, bco2, gstt1, and gstz1. It also revealed significant enrichment in key metabolic pathways (fatty acid, cholesterol, and retinol metabolism) and signaling pathways (PPAR and PI3K-Akt). Furthermore, the expression of multiple solute-carrier family members and apolipoproteins was detected, with notable enrichment in lipid digestion and absorption, cholesterol metabolism, and several key immune-related signaling pathways. These findings provide a theoretical basis for understanding the molecular mechanisms of carotenoid-mediated pigmentation in *L. erythropterus*.

## Simple Summary

*Lutjanus erythropterus* is a key aquaculture species in the South China Sea, and its market value is significantly influenced by body coloration. Juvenile fish were divided into a control group and an astaxanthin-supplemented group to investigate the molecular mechanisms underlying coloration development. The results indicate that astaxanthin promotes growth and development, enhances the activity of liver antioxidant enzymes, and increases skin redness as well as carotenoid content. Furthermore, it influences red body color formation by regulating transcription levels in multiple tissues.

## 1. Introduction

Aquaculture, the farming of aquatic organisms such as fish, mollusks, and crustaceans, is one of the fastest-growing food production sectors in the world [1]. This practice is critically important for global food security and nutrition, providing a primary source of protein for billions of people and alleviating pressure on wild fish stocks [2]. *Lutjanus erythropterus* is an economically important marine species in the South China Sea, characterized by its vivid red body coloration and high nutritional value, which render it highly prized in the market. Its body exhibits a vivid red coloration and possesses high nutritional value, making it highly prized. Due to its rapid growth rate and high market value, the scale of *L. erythropterus* aquaculture has expanded significantly [3]. However, large-scale, high-density farming has led to poor coloration in farmed *L. erythropterus*. Unlike their wild counterparts, farmed specimens cannot adequately consume astaxanthin-rich prey such as shrimp and crabs. When coupled with high-density farming conditions, this significantly impacts the fish’s body coloration, reducing its market value. Notably, the diverse coloration patterns observed in fish arise factors from the complex interplay between genetic background and environmental factors [4].

The development of vibrant body coloration in fish is dependent on carotenoids, which they cannot synthesize endogenously [5]. Therefore, the type and nutritional level of pigments in feed play a critical role in determining fish coloration. In recent years, accumulating research has explored regarding the effects of various dietary additives—such as lipids, proteins, canthaxanthin, and astaxanthin—on fish pigmentation [6,7]. Studies have demonstrated that incorporating ingredients including *Chlorella*, *Haematococcus pluvialis* powder, capsanthin, and *Phaffia rhodozyma* into feed can effectively enhance skin coloration in aquatic species. In an 8-week pigmentation trial on blood Parrot fish (*Cichlasoma synspilum* × *Cichlasoma citrinellum*), Li et al. [8] tested six different carotenoid sources and found that astaxanthin provided the most significant pigmentation enhancement, followed by *H. pluvialis* and *P. rhodozyma*. Consequently, astaxanthin has become a widely adopted coloring agent in aquaculture, valued not only for its remarkable ability to improve skin pigmentation, but also for its prominent roles in promoting growth and enhancing antioxidant capacity [9,10]. Feeding trials with astaxanthin have been conducted in fish species, including *Larimichthys crocea* [11], *Pagrus pagrus* [12], all yielding favorable body coloration outcomes. Genetic makeup serves as a key endogenous factor in color variation, while neuroendocrine regulators are crucial in modulating growth, reproduction, and pigmentation. Even the same external coloration may be governed by distinct genetic mechanisms across different fish species. Advances in genomic technologies have revealed the polygenic nature of fish coloration control [13]. With the maturation of transcriptome sequencing technology, numerous studies have employed this approach to investigate key genes and metabolic pathways associated with fish color development—for instance, in *Oreochromis* sp. [14,15], *Malabar perch* [6], *rainbow trout* [16,17], and *Atlantic salmon* [18]. These studies have identified important genes associated with red phenotypes (e.g., *bco1*, *bco2*, *pax7*, *Stard5*, *CD36*, and *Scarb1*), as well as genes related to melanin synthesis (e.g., *tyr*, *dct*, *mitf*, *pax3a*, *vps11*, and *bmp2*). Pathways linked to melanin synthesis, including PI3K/Akt, α-MSH, and MAPK, were identified, alongside pathways associated with erythromelanin synthesis such as ABC transporters, fatty acid metabolism, retinol metabolism, and cytochrome P450 [19]. However, studies on carotenoid metabolism pathways influencing red coloration in fish remain incomplete. Understanding of key color genes involved in carotenoid pigmentation remains limited, despite existing investigations into intestinal carotenoid absorption and retinol metabolism [20,21,22]. Currently, research on the body color of *L. erythropterus* only includes the study by Chen Zizhao et al. [23] on the early morphology and the developmental sequence of pigment cells, as well as the transcriptome sequencing study of the dorsal skin and ventral red skin by Zhang Yanping et al. [24]. However, there is still a lack of research on the vital internal tissues influencing body color formation in *L. erythropterus*. Therefore, this study measured body color parameters, growth performance, and liver antioxidant enzyme activities in two groups of *L. erythropterus* and integrated transcriptome sequencing of the liver, blood, intestine, and skin to analyze changes in gene expression levels. These findings establish a crucial foundation for systematically elucidating the spatiotemporal dynamics of body color development and its molecular regulatory mechanisms in *L. erythropterus*.

## 2. Materials and Methods

### 2.1. Experimental Design and Sample Collection

Juvenile *L. erythropterus* (body length 4.8 ± 0.32 cm, body weight 3.29 ± 0.17 g) were first acclimated in concrete ponds (4 m × 3 m × 1.5 m) for 7 days. Water conditions during acclimation were as follows: salinity 32 ± 1.93, water temperature 30.5 ± 0.69 °C, and dissolved oxygen > 6.9 mg/L. After acclimation, healthy fish of uniform size were randomly selected and divided into a control group (C) and a treatment group (T), with three technical replicates per group to minimize the effect of individual variation. Each replicate was stocked with 180 fish and reared in experimental concrete pond (4 m × 3 m × 1.5 m). The experimental diet was formulated to contain red fish meal, soybean meal, peanut meal, and corn gluten meal as its primary protein sources, with fish oil and soybean lecithin serving as the main lipid sources (Table 1). All raw materials were procured from Zhanjiang Science Innovation Laboratory Equipment Co., Ltd., Zhanjiang, China. Two experimental diets were formulated: a control diet (A0) containing 0 mg/kg astaxanthin and a treatment diet (A1) containing 200 mg/kg astaxanthin. The diets were prepared as follows: protein ingredients (e.g., soybean meal and peanut meal) were ground to pass through a 60-mesh sieve. The resulting powder was thoroughly mixed with fish oil, soybean lecithin, and water. This mixture was then processed into 3 mm diameter pellets using a TSE65 twin-screw extruder (Beijing Xiandai Yanggong Machinery Technology Development Co., Ltd., Beijing, China). The pellets were dried in a conditioned room for 48 h and subsequently stored at −20 °C. The moisture, crude protein, and crude lipid contents of the raw materials, as well as the nutritional composition of both the control and treatment diets, were determined by Sichuan Weier Testing Technology Co., Ltd., Chengdu, China. Fish were fed twice daily at 08:00 and 16:00 to apparent satiation. During the experiment, 50% of the pond water was periodically exchanged, and aerators were used to maintain adequate dissolved oxygen levels. Water conditions in the experimental ponds were maintained as follows: salinity 32 ± 0.88 (psu), water temperature 29.9 ± 0.47 °C, and dissolved oxygen > 6.9 mg/L. The culture waters were taken every 2 days and tested for ammonia nitrogen and salinity using a portable spectrophotometer (DR1900, HACH, Loveland, CO, USA). Dissolved oxygen was measured using a portable multiparameter water quality analyzer (HQ2100, Hash, CO, USA).

Samples were collected at 4 and 6 weeks into the experiment. Prior to sampling, *L. erythropterus* were anesthetized and euthanized using MS-222 (Sigma, St. Louis, MO, USA). All experimental animals were humanely handled in accordance with China’s regulations on scientific and technological applications. Nine fish were selected from both the control group (C) and treatment groups (T), respectively. Five tissues—blood, intestine, liver, skin, and muscle—were collected from each fish, designated as CBL, CG, CL, CSK (for the control group), and CM, and TBL, TG, TL, TSK, and TM (for the treatment group), respectively. Tissues from three fish were pooled into one sample, placed in RNAase-free cryovials, rapidly frozen in liquid nitrogen, and subsequently stored at −80 °C. Among these, the blood, intestine, liver, and skin samples were sent for transcriptome sequencing.

### 2.2. Fish Growth Indice

Fish were sampled at the end of the temporary culture and week 4 (T4) to measure their growth and physiological parameters. Thirty fish were randomly selected per group, with measurements indices including body length, body weight, weight gain rate (WGR, %), specific growth rate (SGR, %/day), body length growth rate (PLG, %), condition factor (CF), and survival rate (SR, %). The formulas for each growth parameter are as follows [25]:

Weight Gain Rate (WGR, %) = [(W(final) − W(initial))/W(initial)] × 100%

Specific Growth Rate (SGR, %/day) = [(Final live weight − Initial live weight)/t] × 100%

Condition Factor (CF) = Body weight/Body length^3^ × 100%

Survival Rate (SR, %): Number of surviving fish at the end of experiment/Initial number of stocked fish × 100%

Body Length Growth Rate (PLG, %): [(L(final) − L(initial))/L(initial)] × 100%

where W(initial), W(final) represent the initial and final body weights, respectively; t denotes the experimental duration (days); L(initial), L(final) represent the initial and final body lengths, respectively.

### 2.3. Body Color Measurement

Following the color space standards established by the International Commission on Illumination (CIE), each group of experimental fish was anesthetized with MS-222 (Sigma, MO, USA). Surface moisture was blotted dry with absorbent paper. A colorimeter (3nh NR60CP, Guangdong, China) was employed to measure colorimetric values at three locations: the dorsal skin, ventral skin, and gill cover. The L* value represents lightness, with higher values indicating a brighter body color. The a* value denotes red-green chroma:, positive values correspond to a reddish hue, while negative values indicate a greenish hue. The b* value represents yellow-blue chroma: positive values indicate a yellowish hue, and negative values correspond to a bluish hue. A total of 35 samples were measured per group, with two measurements taken at each location. For the second measurement, the colorimeter probe was rotated 180 degrees.

### 2.4. Total Carotenoid Content Extraction and Full-Wavelength Scanning of Various Tissues

A 0.1 g aliquot of each tissue (skin, muscle, liver, intestine, eye, and blood) was weighed and transferred into centrifuge tubes. An equal mass of anhydrous sodium sulfate and 1 mL of acetone were added, followed by tissue homogenization. The homogenate was resuspended in acetone to a final volume of 10 mL and store at 4 °C in the dark for 3 days. Samples were then centrifuged at 4000× *g* for 10 min, and 1 mL of the supernatant was collected for subsequent analysis. Acetone was used as the blank control. Full-wavelength scanning (300–700 nm) was performed on skin tissue samples of *L. erythropterus* skin using a UV spectrophotometer (DR1900, HACH, CO, USA) to identify the maximum absorption peak, which was determined to be 473 nm. The absorbance values of each tissue samples were measured at this specific wavelength.

The total carotenoid content (TCC) was calculated using the formula: TCC (μg/g) = (A × K × V)/(E × G). Where TCC = total carotenoid content (μg/g); A = absorbance value at the maximum absorption peak (473 nm); K = constant (10^4^); V = total volume of the extraction liquid volume (mL); E = molar extinction coefficient (2500), defined as the theoretical absorbance value of a 1 g/L mass concentration solution in a 1 cm pathlength cuvette; G = initial mass of the tissue sample (g).

### 2.5. Determination of Antioxidant Enzyme Activity in Liver

Three liver tissue samples were collected per group. After thawing at 4 °C, 0.1 g of each sample was excised and transferred into a 2 mL centrifuge tube. A total of 1.8 mL of cold 0.86% physiological saline was added, followed by tissue homogenization to prepare a 10% tissue homogenate. The prepared 10% homogenate was centrifuged at 3000× *g* for 10 min at 4 °C using a refrigerated centrifuge. the supernatant was collected for subsequent determination of antioxidant parameter, which included total protein (TP) content, total superoxide dismutase (T-SOD) activity, catalase (CAT) activity, and malondialdehyde (MDA) content. All measurements were performed in accordance with the instructions provided in the commercial reagent kits (Nanjing Jiancheng Biological Engineering Institute, Nanjing, China).

### 2.6. Transcriptome Sequencing and Data Processing

Total RNA was extracted using the Trizol method. RNA concentration and purity were assessed with a NanoDrop 2000 microvolume nucleic acid analyzer (Wilmington, DE, USA), while RNA integrity was precisely evaluated using an Agilent 2100 Bioanalyzer (Santa Clara, CA, USA). The OD260/280 ratio ranged from 2.0 to 2.2, the OD260/230 ratio from 1.8 to 2.0, and the RNA integrity (RIN) exceeded 7.2, confirming that the RNA samples were of sufficient quality for sequencing. Three biological replicates per tissue were used to construct cDNA samples. Sequencing was performed by Wuhan Feisha Gene Co., Ltd., Wuhan, China on the MGI high-throughput sequencer platform. Raw reads were filtered using SOAPnuke software (V2.1.0) to remove adapter-contaminated paired reads, low-quality sequences, and those with a N content (where N denotes unidentified base information) exceeding 0.5%, yielding the final clean reads. The Hisat2 alignment tool (V 2.2.1) was used to map the clean reads from each sequencing sample to the reference genome of *L. erythropterus* (NCBI Taxonomy ID: 211835). Gene expression quantification was performed using FeatureCounts software (V2.4.3). The Trimmed Mean of M-values(TMM) normalization algorithm was employed to calculate gene expression levels across samples, thereby mitigating errors associated with variations in sequencing depth.

### 2.7. Differential Gene Expression Analysis, GO and KEGG Enrichment Analysis

Differential gene expression analysis was performed using DESeq2 software (V1.32.0) with screening thresholds set at |Log2Fold Change| > 1 and q-value < 0.05. Four comparison groups were constructed between the control and treatment groups across the four tissues: CBL vs. TBL, CG vs. TG, CL vs. TL and CSK vs. TSK. Functional annotation of the protein sequence files from the *L*. *erythropterus* genome was conducted using EggNOG-mapper (V2.1.6) to acquire gene-related information. Gene Ontology (GO) and Kyoto Encyclopedia of Genes and Genomes (KEGG) annotation data were extracted based on the gene functional annotations. The OrgDb installation package for *L*. *erythropterus* was constructed using AnnotationForge software (V1.34.1). GO and KEGG enrichment analysis were performed on differential expression gene results for each comparison group using clusterProfiler software (V4.0.5).

### 2.8. RT-qPCR

To validate the accuracy of the transcriptome data, ten differentially expressed genes (Table 2) were randomly selected for RT-qPCR validation. Gene-specific primers were designed using Primer Premier v5.0. software, with *rab10* serving as the internal reference gene. The relative transcript levels of each gene were calculated using the 2^-ΔΔCt^ method and subjected to statistical analysis. Results from both RT-qPCR and RNA-seq were visualized using GraphPad Prism v8.0.2.

### 2.9. Statistical Analysis

Data are presented as mean ± standard deviation and were analyzed using one-way ANOVA (SPSS 22.0) followed by Duncan’s multiple comparisons test. Supplementary verification was performed using Student’s *t*-test, with *p* < 0.05 considered statistically significant. All graphs were generated using GraphPad Prism 8.0.

## 3. Results

### 3.1. The Effect of Astaxanthin on the Growth Performance of L. erythropterus

The growth performance of *L. erythropterus* is shown in Table 3. The treatment group fed with astaxanthin exhibited superior growth performance compared to the control group. After 4 weeks of the experiment, the treatment group exhibited significantly higher final body length, final body weight, body length growth rate, weight gain rate, and specific growth rate compared to the control group (*p* < 0.05). No significant effect of astaxanthin supplementation was observed on the condition factor.

### 3.2. The Effect of Astaxanthin on Color Change of L. erythropterus

**Figure 1:**
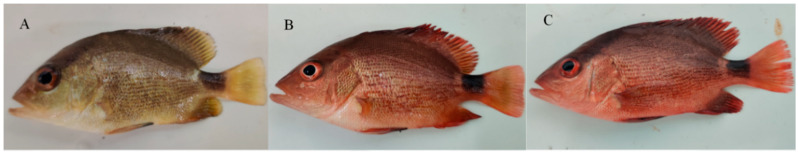
Differences in body coloration of *L. erythropterus* among control group for 4 weeks ((**A**): C4), treatment group for 4 weeks ((**B**): T4), and treatment group for 6 weeks ((**C**): T6).

As shown in Figure 1, the skin color of the astaxanthin-supplemented treatment groups was redder than that of the control group. As indicated in Table 4, the red-green value a* of the ventral skin, dorsal skin, and gill cover in the T4 and T6 of *L. erythropterus* was significantly higher (*p <* 0.05) than that of the C4. Regarding the lightness value L*, the L* values of the sides and gill covers of *L. erythropterus* in both the T4 and T6 were significantly higher (*p <* 0.05) than those of the C4, while no significant difference was observed on the dorsal skin. For the yellow-blue value b*, both the T4 and T6 showed significantly higher values (*p* < 0.05) on the ventral skin and gill cover compared to the C4, while no significant difference was observed on the dorsal skin.

### 3.3. The Effect of Astaxanthin on Total Carotenoid Content in Various Tissues of the L. erythropterus

**Figure 2:**
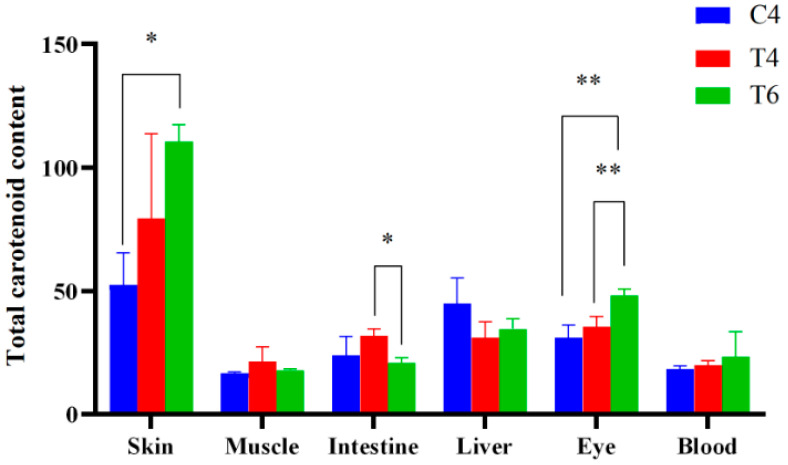
Comparison of total carotenoid content in different groups of *L. erythropterus*. * indicates a significant difference (*p* < 0.05); **: Significantly different (*p* < 0.01).

As presented in Figure 2 and Table 5, the total carotenoid content(TCC) in various tissues of *L*. *erythropterus* fed the astaxanthin-enriched diet was higher than that in the control group, and this content increased with extension of feeding duration. The skin TCC of *L. erythropterus* in the T6 was significantly higher than that in C4 group (*p* < 0.05). The intestinal TCC of fish in the T4 group was significantly higher than that in the T6 group. Additionally, the ocular TCC of fish in the T6 group was significantly higher than that in both the C4 and T4 groups (*p* < 0.05). As indicated in Table 4, the skin exhibited the highest TCC, which was significantly higher than that of other tissues (*p* < 0.05). The ranking of TCC from highest to lowest across groups was as follows: C4 group: Skin > Liver > Eye > Intestine > Blood > Muscle; T4 group: Skin > Eye > Intestine > Liver > Muscle > Blood; T6 group: Skin > Eyes > Liver > Blood > Intestine > Muscle.

### 3.4. The Effect of Astaxanthin on Antioxidant Enzyme Activity in the Liver of the L. erythropterus

**Figure 3:**
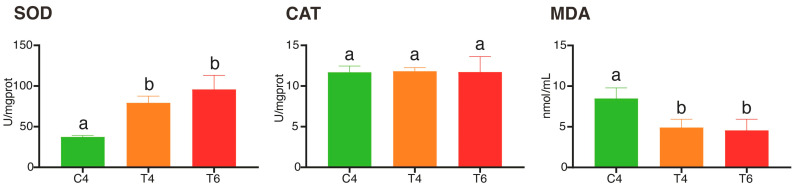
Comparison of antioxidant capacity (SOD, CAT and MDA) in the liver of different groups of *L. erythropterus*. Note: In the table, different lowercase letters in the same row indicate significant differences (*p* < 0.05), while the same lowercase letters indicate insignificant differences (*p* > 0.05). C4: Control group for 4 weeks; T4: Treatment group for 4 weeks; T6: Treatment group for 6 weeks.

This study evaluated the antioxidant capacity of liver tissue in *L. erythropterus* from the *control group* and *treatment groups*, with the results presented in Figure 3. The liver T-SOD activity in the T4 and T6 groups was significantly higher than that in the control group, while no significant difference in CAT activity was observed among all groups. Additionally, the MDA content in the control group was significantly higher than in the treatment groups.

### 3.5. Transcriptome Sequencing Results and Reference Genome Alignments

This experiment performed transcriptomic sequencing on the treatment group (T) and control group (C). As shown in Table 6, the Q30 percentage ranged from 91.7% to 93.8%, with an average GC content of 47.62%. The average reference genome alignment rate across all samples was 89.38%, and the average gene expression quantification rate was 54.35%. These results demonstrate that the transcriptomic sequencing data are of high quality and suitable for subsequent analyses.

### 3.6. Differentially Expressed Gene Analysis

**Figure 4:**
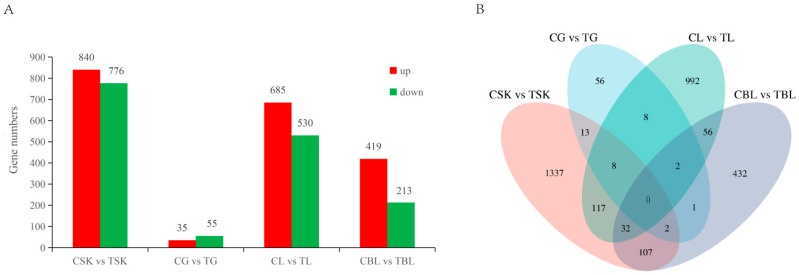
Analysis of transcriptome differential genes in different tissues of *L. erythropterus*. Note: (**A**): Comparison of different transcriptome genes in different tissues; (**B**): Venn map of different genes in different tissues.

Differentially expressed genes (DEGs) were analyzed in four carotenoid metabolism-related tissues (skin, intestine, liver, and blood) between the control and treatment groups of *L. erythropterus*. The results are shown in Figure 4. In the skin tissue, 1616 DEGs were identified between the two groups, with 840 upregulated and 776 downregulated in control group. In the intestine, 90 DEGs were identified between the control and treatment groups, including 35 upregulated and 55 downregulated DEGs in the control intestine. For the liver tissue, 1215 DEGs were identified between the two groups, with 685 upregulated and 530 downregulated in the control group. In blood samples, 632 DEGs were found between the control and treatment groups, including 419 upregulated and 213 downregulated DEGs in the control group.

### 3.7. GO Enrichment Analysis

**Figure 5:**
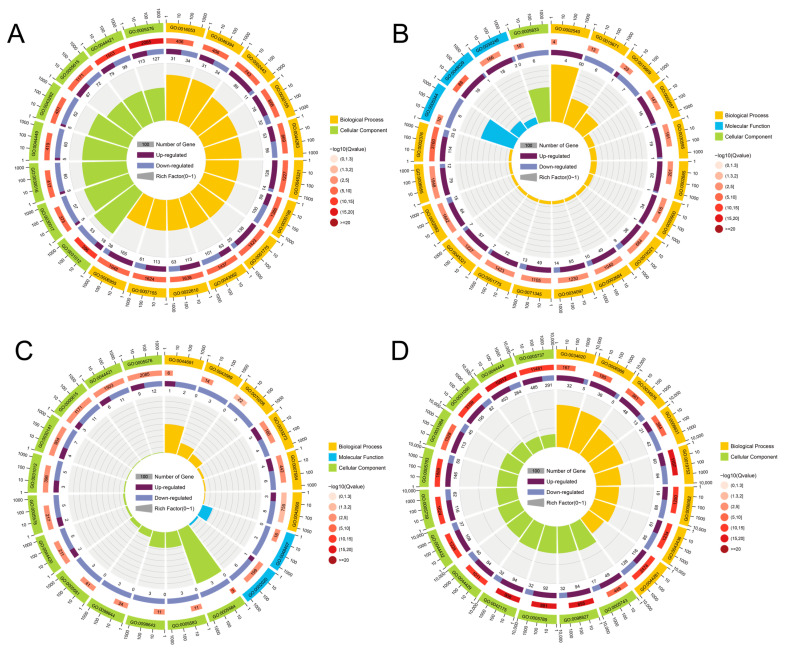
Classification of GO enrichment function of differential genes. Note: (**A**): skin; (**B**): blood; (**C**): intestine; (**D**): liver.

As presented in Figure 5, transcriptomic analysis of four carotenoid-metabolizing tissues (skin, intestine, liver, and blood) identified tissue-specific differentially expressed genes (DEGs) between the control and treatment groups. Using the threshold of q < 0.05, the skin showed 1616 DEGs, which were enriched in 657 Gene Ontology (GO) terms, predominantly in biological processes (BP) and cellular components (CC); The intestine contained 90 DEGs, mapped to 41 GO terms across BP, CC, and molecular function (MF); The liver exhibited 1215 DEGs enriched in 436 GO terms, mainly in BP and CC; The blood revealed 632 DEGs, assigned to 162 GO terms spanning all three GO categories.

### 3.8. KEGG Enrichment Analysis

**Figure 6:**
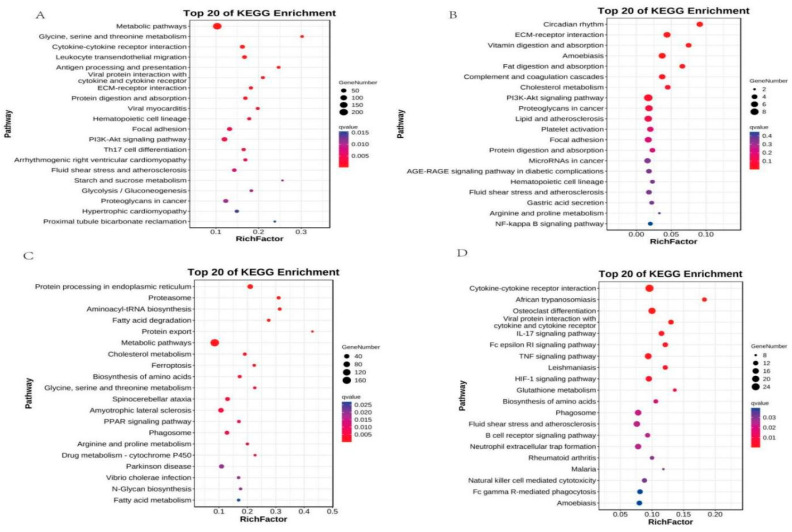
Top 20 KEGG enrichment pathways in each organization. Note: (**A**): skin; (**B**): intestine; (**C**): liver; (**D**): blood. The size of the black circle indicates the number of genes; the redder the color, the greater the significance.

To explore pathway-level differences, differential transcriptome analysis was conducted across four key carotenoid-metabolizing tissues, with a focus on pathways including Protein processing in endoplasmic reticulum and Proteasome. KEGG enrichment analysis was performed on the identified DEGs. The KEGG pathway database classifies biological metabolic processes into six major classes: cellular processes, environmental information processing, genetic information processing, human disease, metabolism, and organismal systems. KEGG enrichment results showed the following (Figure 6): DEGs in skin, intestine, liver, and blood were enriched in 333, 171, 326, and 308 KEGG pathways, respectively; Among skin-specific DEGs, major enrichment occurred in metabolic pathways, glycine, serine, and threonine metabolism, ECM-receptor interaction, PI3K-Akt signaling pathway, and glycolysis/gluconeogenesis. In intestinal DEGs, enrichment was observed in ECM-receptor interaction, vitamin digestion and absorption, fat digestion and absorption, cholesterol metabolism, PI3K-Akt signaling pathway, and MAPK signaling pathway; In the liver, DEGs were significantly enriched in pathways including fatty acid degradation, protein export, metabolic pathways, and PPAR signaling. In contrast, DEGs in blood were primarily associated with cytokine-cytokine receptor interaction, IL-17 signaling, TNF signaling, HIF-1 signaling, and glutathione metabolism.

### 3.9. Validation with RT- qPCR

**Figure 7:**
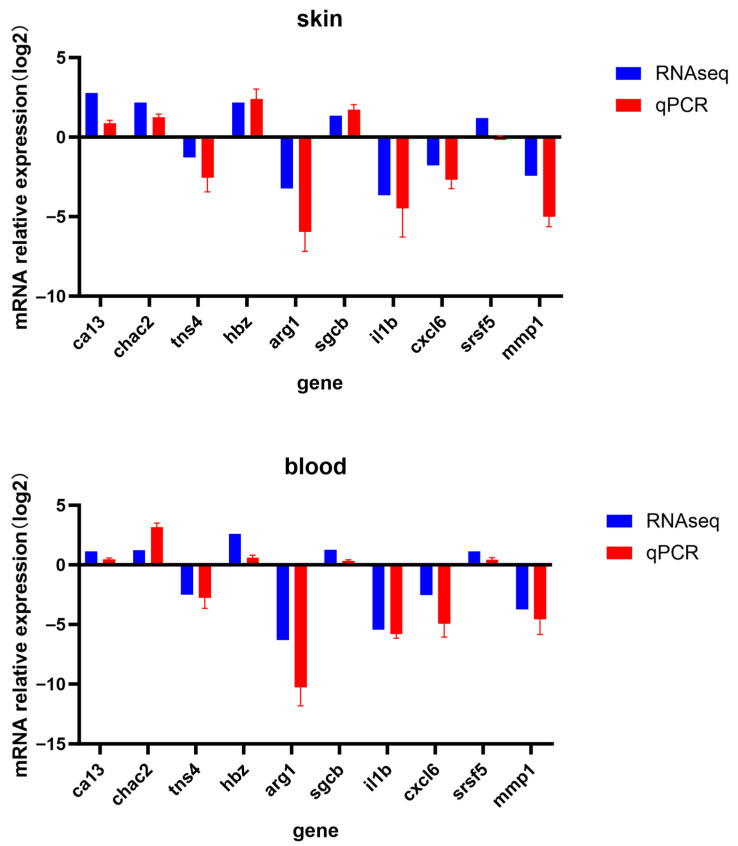
RT-qPCR verification of some differential genes in *L. erythropterus*.

To further validate the accuracy and reliability of our transcriptomic data, ten differentially expressed genes (DEGs) were randomly selected—five up-regulated and five down-regulated—and assessed their relative expression levels were quantified in the skin and blood using reverse transcription quantitative polymerase chain reaction (RT-qPCR). Comparative analysis revealed that the gene expression patterns detected by RT-qPCR were consistent with those from transcriptomic sequencing data (Figure 7). This result confirms the reliability and accuracy of the RNA sequencing (RNA-seq)-based transcriptomic expression profiles generated in this study.

## 4. Discussion

Fish coloration is primarily dictated by the type and abundance of chromatophores in the dermal layer, which vary significantly throughout development [26,27]. Four types of chromatophores have been identified in *L. erythropterus*. The characteristic bright red body color of adult individuals is a result of the pronounced dominance of erythrophores. This red pigmentation is dependent on carotenoids. Notably, fish cannot synthesize carotenoids de novo and thus rely entirely on dietary intake [28]. In this experiment, *L. erythropterus* were fed a diet supplemented with astaxanthin. After 4 and 6 weeks, the astaxanthin-treated group showed significantly higher a* (redness) values on the ventral skin, dorsal skin, and gill covers compared to the control group (*p <* 0.05). Additionally, the L* (lightness) and b* (yellowness) values on the lateral skin and gill covers were significantly increased (*p* < 0.05), confirming that astaxanthin enhances skin redness in this species. Subsequently, total carotenoid content was analyzed in multiple tissues of both the treatment and control groups. Our results demonstrate that the skin accumulated significantly more astaxanthin than any other tissues (*p* < 0.05), as illustrated in Figure 2. Notably, the eyes and liver showed intermediate concentrations, while muscle had the lowest and skin had the highest, identifying the skin as the predominant site for carotenoid deposition. Jiang et al. [29] investigated the color-enhancing effects of astaxanthin on koi carp (*Cyprinus carpio*) across different feeding durations. At an astaxanthin dosage of 130 mg/kg, they observed that total carotenoid content in the skin exhibited significant changes over time, while pigment deposition in the eyes, hepatopancreas, and muscle showed minimal variation. The skin exhibited the highest total carotenoid content, followed by eyes and hepatopancreas, with the muscle showing the lowest levels. This finding is consistent with the pattern of pigment deposition we observed in *L. erythropterus*.

Astaxanthin has garnered significant attention due to its remarkable antioxidant and pigmentation-enhancing properties. It has been developed not only as a health supplement in the pharmaceutical market, but has also been wildly applied in aquaculture [30,31,32]. This study found that astaxanthin enhances growth performance in *L. erythropterus*. After four weeks of cultivation, the treatment group exhibited significantly greater final body length, final body weight, weight gain rate, length growth rate, and specific growth rate compared to the control group (*p* > 0.05). This indicates astaxanthin promotes growth and development in *L. erythropterus*, consistent with most existing research findings [10,32,33]. Previous studies have also demonstrated that astaxanthin can enhance fish growth, likely by strengthening the body’s antioxidant system [34,35]. As a potent exogenous antioxidant, astaxanthin enhances antioxidant defense by activating the Nrf2-ARE pathway and inhibiting NF-κB signaling, thereby increasing the activities of enzyme such as SOD and reducing MDA levels [10]. In this present study, dietary astaxanthin significantly increased total superoxide dismutase (T-SOD) activity and decreased malondialdehyde(MDA) content in the liver of *L. erythropterus* (*p* < 0.05), though catalase (CAT) activity remained unchanged. These results confirm the antioxidant effect of astaxanthin in *L. erythropterus*. Furthermore, some studies indicate that the antioxidant benefits of astaxanthin in fish are conditional. it can enhance antioxidant capacity only when fish are subjected to oxidative stress (e.g., oxidative damage or high stocking density) [36,37]. Therefore, the practical application of astaxanthin in aquaculture should be tailored to specific conditions.

Currently, most molecular studies on fish coloration focus primarily on transcriptomic sequencing analysis of a single tissue—the skin [38]. Angelico et al. [39] extended the scope to other tissues, highlighting that the skin, intestine, liver, and blood are crucial sites for carotenoid metabolism and play significant roles in body color regulation. In this study, comparative transcriptomics across multiple tissues was used to investigate the significant effects of astaxanthin on carotenoid metabolic pathways in the skin, intestine, liver, and blood of *L. erythropterus*. Differentially expressed genes were enriched in pathways related to body coloration, lipid metabolism, and immune regulation. Multiple genes involved in carotenoid metabolism were identified among the differentially expressed genes in the skin transcriptome. Key enzymes such as carotenoid oxygenases *bco1* and *bco2*, critical to skin and flesh coloration, were significantly upregulated in the astaxanthin-treated group. Several lipid transport proteins were also differentially expressed. Genes including *gstt1*, *apobec2*, *fabp2*, *gstz1*, and *rpe65* were significantly upregulated, while *vldlr* and *apoe* were downregulated. *gstt1* and *gstz1*, belonging to the glutathione S-transferase family, may function similarly to *gsta2*, which is associated with carotenoid-based pigmentation, *RPE65*, a member of the carotenoid oxygenase superfamily, participates in retinoid metabolism and carotenoid isomerization [40]. Additionally, several short-chain dehydrogenase/reductase (SDR) genes (*dhrs7c*, *dhrs13*, and *dhrs1)* were significantly overexpressed in the treatment group. These enzymes facilitate retinol and retinaldehyde redox reactions and have been implicated in carotenoid-related red pigmentation in other fish species, suggesting a shared role in color formation [41].

The intestine is the primary site for astaxanthin absorption. Upon digestion, dietary carotenoids are released and taken up by intestinal epithelial cells via receptors such as SCARB1 and CD36 [42]. The enzymes *bco1* and *bco2*, expressed in various tissues including the intestine, cleave and convert carotenoids to maintain metabolic balance [43,44,45]. After absorption, carotenoids are packaged into chylomicrons and transported to the liver for storage as retinyl esters, which bind to retinol-binding proteins [21]. In our study, hepatic expression of *scarb1* was upregulated in the treatment group, along with other key genes involved in carotenoid metabolism (*abcg8*, *abca4*, *gstz1*, *elovl1*, *elovl5*, *lart*). Several cytochrome P450 genes (*cyp1c1*, *cyp2w1*, *cyp4b1*, *cyp20a1*, *cyp7a1*), crucial for liver detoxification, were also identified as differentially expressed. These genes play crucial roles in liver detoxification and the metabolism of exogenous substances, and be important for the formation of red body coloration in *L. erythropterus*. As the primary carotenoid transport medium, blood distributes dietary astaxanthin to the liver and subsequently to peripheral tissues. Our differential expression analysis in blood identified upregulation of multiple transporter genes in response to astaxanthin supplementation, implying their involvement in pigment transport. Notably, the strong upregulation of monophenol monooxygenase suggests a potential interaction between astaxanthin and melanin-related metabolism. This was corroborated by KEGG enrichment, which highlighted pathways for both melanin metabolism and red pigmentation—including arachidonic acid metabolism, a known pathway for red coloration in related species [41]. Thus, astaxanthin influences body color by coordinating transport and metabolic processes. In summary, astaxanthin feeding significantly influenced carotenoid metabolic pathways at the transcriptome level in tissues including the skin, intestine, liver, and blood of *L. erythropterus*. Differentially expressed genes were enriched in pathways related to body coloration, lipid metabolism, and immune regulation, affecting the mRNA expression levels of multiple color-related genes. This indicates that 200 mg/kg astaxanthin can influence the formation of red body coloration by affecting the transcriptional levels in various tissues of *L. erythropterus*.

## 5. Conclusions

This study confirms that dietary supplementation with 200 mg/kg astaxanthin significantly promotes growth and enhances hepatic antioxidant capacity in *L. erythropterus*, as evidenced by increased T-SOD activity, reduced MDA content, and unaffected CAT activity. Additionally, astaxanthin markedly improved skin redness and total carotenoid content, with the highest deposition observed in the skin. Transcriptomic analysis revealed that astaxanthin modulates the expression of genes involved in pigment metabolism, lipid metabolism, and immune-related pathways across multiple tissues—including skin, intestine, liver, and blood—thereby influencing red coloration. These results demonstrate that 200 mg/kg astaxanthin effectively promotes the development of red body color in *L. erythropterus*.

## Figures and Tables

**Table 1:** Feed formula and nutrient composition (%).

Ingredient	A0	A1
Red fish meal	71	71
Soybean meal	6	6
Peanut meal	5	5
Corn gluten powder	5	5
Bread flour	2	2
Fish oil	5	5
Soybean lecithin	3.5	3.5
Ca(H_2_PO_4_)_2_	1	1
Mineral premix ^a^	0.5	0.5
Feeding attractant	0.05	0.05
Vitamin C	0.05	0.05
Antioxidants	0.05	0.05
Choline chloride	0.5	0.5
Microcrystalline cellulose	0.35	0.15
(10%) Astaxanthin ^b^	0	0.2
Total	100	100
Nutrient levels		
Moisture	10.1	10.7
Crude protein	53.06	52.54
Crude fat	11.1	11.2
Ash	14.3	14.7

Note: a: Premix (contained in each kilogram of this product): vitamin A ≥ 300,000 IU, vitamin B2 ≥ 0.45 g, vitamin B6 ≥ 0.15 g, vitamin B12 ≥ 0.0009 g, vitamin C ≥ 0.0 g, vitamin D3 ≥ 35,000 IU, vitamin K3 ≥ 0.08 g, nicotinamide ≥ 2.0 g, biotin ≥ 0.004 g, calcium pantothenate ≥ 0.9 g, copper ≥ 3.0 g, iron ≥ 30.0 g, zinc ≥ 20.0 g, manganese ≥ 2.4 g, moisture ≤ 10%. b: Ludingkang Pink BASF 10% Astaxanthin: BASF GmbH, Ludwigshafen, Germany.

**Table 2:** Primer sequences for RT-qPCR amplification of genes in *L. erythropterus*.

Gene	Primer (5′-3′)
*rab10*	F: GAGGGTCGTACCAAAAGCCA
	R: GTTGGCCTTAGCACTCGTCT
*arg1*	F: ATCGGCTCCATCCACGGTCAC
	R: ACACCTTCACACCCAGGAGCTT
*il1b*	F: AAAACCTGCTCAACATCATGCT
	R: GTTAGTTCCTTCACTGCCTCCC
*mmp1*	F: CATCGCCAGTTTCTCCACGTT
	R: CGCTGTAGATCCTTGTGAACCTC
*cxcl6*	F: GCTGATTCTGCCTAACTCACAC
	R: GACTTTCTTCACCCAGGGAGC
*tns4*	F: GACTGATATTCCTGTGCTGCT
	R: AATGTTCCTGCTGTCTTGTCC
*srsf5*	F: ACTTGTCCTCTCGTGTCAGC
	R: ACTTCTCGACCTCTTCTTGGC
*hbz*	F: GACCAAGACTTACTTCGCCCACT
	R: AGCAGCCAGAAACTTGTCCAC
*ca13*	F: CCAACCCCAGGATTCAGAGAGT
	R: AGCCTCTCCTTCTGCAGTGA
*chac2*	F: ATCGGCTACATTAAAGGCTTC
	R: CCGTGATGACCTGATAACCAC
*sgcb*	F: ACTACACAAGAGCACCGTA
	R: TCCCCTTTAATGTTCAGGTCA

**Table 3:** Effects of astaxanthin on the growth of *L. erythropterus*.

Parameter	C4	T4
Initial body length/cm	4.81 ± 0.32	4.81 ± 0.32
Initial weight/g	3.29 ± 0.17	3.29 ± 0.17
Final body length/cm	6.76 ± 0.38 ^a^	7.57 ± 0.67 ^b^
Final weight/g	5.59 ± 0.96 ^a^	8.21 ± 2.35 ^b^
Body length growth rate/% PLG	16.85 ± 6.71 ^a^	31.01 ± 11.72 ^b^
Weight gain rate/% WGR	69.88 ± 29.52 ^a^	149.46 ± 72.41 ^b^
Specific growth rate/(%/d) SGR	1.84 ± 0.61 ^a^	3.13 ± 0.98 ^b^
Condition factor CF	3.13 ± 0.30	3.20 ± 0.18
Survival rate/% SR	90.0	95.6

Note: In the table, different lowercase letters in the same row indicate significant differences (*p* < 0.05), while the same lowercase letters indicate insignificant differences (*p* > 0.05). C4: Control group for 4 weeks; T4: Treatment group for 4 weeks. The same in the table below.

**Table 4:** Effect of astaxanthin on skin chroma value of *L. erythropterus*.

Location	Chromaticity Value	C4	T4	T6
Ventral skin	L*	77.86 ± 20.75 ^a^	82.30 ± 9.65 ^b^	85.55 ± 6.27 ^b^
	a*	−5.97 ± 10.47 ^a^	12.76 ± 5.81 ^b^	16.30 ± 4.54 ^b^
	b*	1.48 ± 18.92 ^a^	19.70 ± 6.61 ^b^	19.52 ± 6.73 ^b^
Dorsal skin	L*	69.55 ± 4.60 ^a^	62.30 ± 4.98 ^a^	61.92 ± 3.10 ^a^
	a*	3.33 ± 0.94 ^a^	4.34 ± 1.29 ^b^	5.46 ± 1.37 ^b^
	b*	9.58 ± 2.22 ^a^	6.31 ± 1.72 ^a^	7.04 ± 1.35 ^a^
Gill cover	L*	14.39 ± 5.52 ^a^	54.59 ± 37.19 ^b^	40.00 ± 39.76 ^b^
	a*	−90.83 ± 20.42 ^a^	−17.08 ± 37.92 ^b^	−30.30 ± 44.15 ^b^
	b*	−23.08 ± 11.99 ^a^	−0.71 ± 28.82 ^b^	−12.06 ± 32.60 ^b^

Note: In the table, different lowercase letters in the same row indicate significant differences (*p* < 0.05), while the same lowercase letters indicate insignificant differences (*p* > 0.05). C4: Control group for 4 weeks; T4: Treatment group for 4 weeks; T6: Treatment group for 6 weeks.

**Table 5:** Effect of astaxanthin on total carotenoid content in tissues of *L. erythropterus*.

Tissue	C4	T4	T6
Skin	52.38 ± 10.71 ^a^	79.30 ± 28.16 ^a^	110.62 ± 5.48 ^a^
Muscle	16.72 ± 0.26 ^c^	21.40 ± 4.81 ^b^	17.89 ± 0.57 ^d^
Intestine	23.94 ± 6.17 ^c^	31.87 ± 2.29 ^b^	20.85 ± 1.70 ^d^
Liver	44.90 ± 8.35 ^ab^	31.05 ± 5.26 ^b^	34.64 ± 3.24 ^c^
Eyes	31.07 ± 4.16 ^bc^	35.45 ± 3.43 ^b^	48.17 ± 2.10 ^b^
Blood	18.40 ± 0.98 ^c^	19.87 ± 1.60 ^b^	23.46 ± 8.22 ^d^

Note: In the table, different lowercase letters in the same column indicate significant differences (*p* < 0.05), while the same lowercase letters indicate insignificant differences (*p* > 0.05).

**Table 6:** Statistical analysis of transcriptome sequencing results.

Samples	Clean Reads	Clean Bases (G)	Effective Rate (%)	Q30	GC Content (%)
CBL-1	72,913,062	10.94	93.53	91.8	49.0
CBL-2	77,026,538	11.55	94.06	92.0	49.3
CBL-3	80,300,150	12.05	93.73	91.7	49.0
CG-1	61,788,340	9.27	90.70	93.6	46.7
CG-2	59,065,970	8.86	94.34	93.3	46.6
CG-3	70,387,004	10.56	95.43	92.1	47.2
CL-1	73,115,202	10.97	95.34	93.8	46.7
CL-2	74,479,514	11.17	95.08	93.3	46.8
CL-3	73,981,624	11.10	94.05	93.7	46.7
CSK-1	5,7442,268	8.62	94.68	92.9	46.9
CSK-2	56,544,130	8.48	94.64	92.1	47.5
CSK-3	72,659,662	10.90	91.54	92.5	47.8
TBL-1	64,366,828	9.66	92.98	91.6	49.3
TBL-2	69,536,200	10.43	94.85	91.7	49.4
TBL-3	79,400,088	11.91	92.55	91.7	49.6
TG-1	53,779,626	8.07	95.16	92.9	47.0
TG-2	62,145,380	9.32	95.31	92.9	47.2
TG-3	61,689,482	9.25	94.35	92.5	46.4
TL-1	65,672,548	9.85	92.42	92.9	45.8
TL-2	73,566,852	11.04	93.72	92.6	47.1
TL-3	73,708,750	11.06	95.50	91.9	47.7
TSK-1	51,675,468	7.75	96.48	92.0	48.3
TSK-2	60,077,824	9.01	86.32	93.2	46.8
TSK-3	53,900,786	8.09	94.14	92.5	48.0

## Data Availability

The data have been deposited in the China National GeneBank DataBase (CNGBdb) with the Submission ID: SUB073158.
